# How Do Ants Make Sense of Gravity? A Boltzmann Walker Analysis of *Lasius niger* Trajectories on Various Inclines

**DOI:** 10.1371/journal.pone.0076531

**Published:** 2013-10-29

**Authors:** Anaïs Khuong, Valentin Lecheval, Richard Fournier, Stéphane Blanco, Sébastian Weitz, Jean-Jacques Bezian, Jacques Gautrais

**Affiliations:** 1 Centre de Recherche sur la Cognition Animale, Université de Toulouse, UPS, Toulouse, France; 2 The French National Centre for Scientific Research, CRCA, Toulouse, France; 3 Laboratoire Plasma et Conversion d'Energie, Université de Toulouse, UPS, Toulouse, France; 4 The French National Centre for Scientific Research, Laboratoire Plasma et Conversion d'Energie, Toulouse, France; 5 Centre de Recherche d'Albi en Génie des Procédés des Solides Divisés, de l'Energie et de l'Environnement, Université de Toulouse, Mines Albi, France; 6 The French National Centre for Scientific Research, Centre de Recherche d'Albi en Génie des Procédés des Solides Divisés, de l'Energie et de l'Environnement, Albi, France; Arizona State University, United States of America

## Abstract

The goal of this study is to describe accurately how the directional information given by support inclinations affects the ant *Lasius niger* motion in terms of a behavioral decision. To this end, we have tracked the spontaneous motion of 345 ants walking on a 0.5×0.5 m plane canvas, which was tilted with 5 various inclinations by 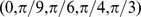 rad (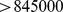 data points). At the population scale, support inclination favors dispersal along uphill and downhill directions. An ant's decision making process is modeled using a version of the *Boltzmann Walker* model, which describes an ant's random walk as a series of straight segments separated by reorientation events, and was extended to take directional influence into account. From the data segmented accordingly (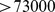 segments), this extension allows us to test separately how average speed, segments lengths and reorientation decisions are affected by support inclination and current walking direction of the ant. We found that support inclination had a major effect on average speed, which appeared approximately three times slower on the 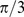 incline. However, we found no effect of the walking direction on speed. Contrastingly, we found that ants tend to walk longer in the same direction when they move uphill or downhill, and also that they preferentially adopt new uphill or downhill headings at turning points. We conclude that ants continuously adapt their decision making about where to go, and how long to persist in the same direction, depending on how they are aligned with the line of maximum declivity gradient. Hence, their behavioral decision process appears to combine klinokinesis with geomenotaxis. The *extended Boltzmann Walker* model parameterized by these effects gives a fair account of the directional dispersal of ants on inclines.

## Introduction

The goal of the present study is to describe accurately the effect of support inclination on the ants *Lasius niger*'s motion in terms of their behavioral decisions, namely how the directional information given by the graviception continuously affects their decision process about where to go. To address this question, we have gathered high-quality movement data by tracking the spontaneous motion of 345 ants walking on a 0.5×0.5 m plane canvas, which was tilted with various inclinations 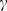 by 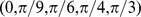 rad.

This study fits in a series of works devoted to the modeling of collective building processes in social insects [1], [2]. Such processes require that individuals (ants, termites) transport tiny loads of material from one place to another. In such a description, an individual picks up a load of material at some place, walks for a while, and ends up dropping its load some distance away from the picking site. Following the stigmergy principles defined by Grassé in the context of nest building in termites [3], the regulation of the final structure is achieved through amplification mechanisms [4], [5]. For instance, in corpse aggregation [1], ants pick and carry corpses around and are more prone to drop their load in places where many corpses were dropped before, so that the more corpses there are at some place, the more additional corpses will be dropped there. In the end, this amplification process leads to the formation of corpse aggregates. More generally, the building of social insect nests, such as ants and termites, emerge from the accumulation of numerous individual transports. Hence, a full description of the individual transports requires the identification of the local decision of picking/dropping a load of material, as well as a detailed description of the paths taken by individuals.

A methodology for modeling this kind of processes has been thoroughly reviewed in a previous article [6], especially for the identification of the behavioral rules governing the individual dropping/picking rates depending on the local conditions. In these studies, building behavior happened on the two-dimensional plane, and ants' motion has so far been modeled using the classical model of diffusion. Diffusion refers to the dynamics of the density of ants as a function of location and time, so it is a macroscopic description of what happens at the population scale. At the individual scale, various *random walk* models have been proposed in biology to describe the movements of animals [7], [8]. For instance, in an experiment reported by [9], *Lasius niger* ants distributed their search efforts over all parts of the experimental area, in the absence of food. Hence, on the level ground and in a pure exploratory context, *Lasius niger* ants' motion is likely to be well described using a random walk. Most terrestrial ants display search behavior based on some kind of random walk, with significant inter-specific differences likely linked to functional concerns with food spotting efficiency [10]. Searching behavior is also known to be affected by internal and/or external factors, from locomotory patterns to external guidelines [11], which can have large scale consequences through colonial amplification in the presence of food [12]. Random walk models can be seen as algorithms which describe the decision making process of a given animal all along its path and produce individual trajectories. Since the term *random walk* refers to different models of the decision process, we restate in the Methods the full details of the version that has been most often used in ants: the *Boltzmann Walker* model. In this description, the path of an animal is approximated in a series of straight moves of various lengths, separated by turning angles. This stochastic model describes how long an animal will move straight ahead, and the choice of the new direction it takes when turning. In every study on a horizontal plane, its parameters measured at the individual scale were shown to yield predictions compatible with typical measures of the diffusive behavior of ants (esp. the diffusion coefficient) [1], [6], [13]–[17].

Now, motivated by the need to progress towards an explanation of 3-dimensional structure construction (termites mounds, ants nests), we need to consider the major difference between motion on a horizontal plane and motion on a tilted surface developing in 3 dimensions, that is, the local inclination of the surface. In the building phase, the tilted and curved surfaces of the structure in progress are expected to modify the ants moving decisions and might thus have in turn an effect on the nest architecture itself [18]. If ants react to support inclination by preferentially adopting some paths, the diffusive model would no longer hold, the whole process of material displacement would be affected, and may produce in turn a different final structure. For instance, Robinson et al. found a slope-based decision in *Pheidole ambigua* dropping their excavated load of soil near the nest entrance, driven by changes of direction preferentially downhill than to uphill. As a consequence, dropping sites are more often located where the slope is the least steeply uphill from the nest entrance, which affects in turn the shape of the ring-shaped pile around the nest entrance [19].

Numerous studies in insects show that the inclination of the support has indeed a strong effect on individual locomotion behavior. For instance, the speed of adult beetles decreased with an increase in the slope of the substrate as a reaction to the increased gravitational force vector opposing uphill movement [20]. In ants, Weihmann & Blickhan advocate that proprioceptive sensing mechanisms, such as graviception, are in regular demand for ants' navigation inside the nest, since sensory stimuli used for foraging outside are lacking [21] and the pheromone-based navigation may be of poor directionality since the inner walls are only passively coated by cuticles' hydrocarbons [22]. In termites *Hospitalitermes rufus*, *H. sharpi*, and *Macrotermes carbonarius*, Jander et al. [23] have found that the orientation angle between the slope direction upward or downward and the direction of walking decreases with increasing slope inclination (geomenotaxis). They suggest that body weight mediates much, if not all, of the gravity perception. The studies dealing with ants on inclines mainly focused on the slope-detection mechanisms, that is how they detect a slope from a biomechanical point of view [24]–[26] or how sensitive this detection is in the context of learning and path integration, especially in the desert ant *Cataglyphis fortis* [27] because path integration along an undulating terrain requires ants to compute the ground projection of their path with sufficient accuracy [28]. Wintergest et al. reported that desert ants are able to discriminate a steeper test slope that differed from the training slope by 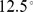 for moderate slopes below 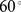 inclination [27]. In those previous studies, the effect of the inclination was measured in set-ups in which the ants were constrained to move along one dimension, either uphill or downhill. To our knowledge, no proposition has been made so far of a full 2-dimensional algorithmic model of the decision-making process in ants moving on an inclined surface without constraints, thereby allowing movement in any directions.

The first step of our analysis was to check that the trajectories of *Lasius niger* are indeed affected when the support is inclined. The section *Experimental Results* report some measures showing this global effect on the statistics of locations, headings and speeds of the ants at the population scale.

To understand this global effect in terms of individual decision processes along the trajectory, we then proceed with the Boltzmann Walker framework. First, we check that this model is still relevant in the present context when ants move on the horizontal plane with no orientation field, and allows the quantitative correspondence between the individual parameters estimated from the trajectories and the population dispersal. We take this level plane condition as the reference case to test for inclination effects.

Then, we consider how precisely the inclination should affect the decision process. Organisms orient themselves to the effect of stimuli (such as heat, light, humidity, gravity etc.) in two ways. One is by a directed orientation reaction (taxis), in which the direction of motion of the organism is influenced by the stimulus. The other method of orientation is an undirected locomotory reaction (kinesis) in which the average speed or the average rate of turning of the organism, but not the direction in which it moves, are dependent on the stimulus [8], [29], [30]. In the diffusive version, there is no directional information that would orient the trajectories of the animals, and the standard BW model takes accordingly for granted that speed, turning rates and reorientation decisions are constant parameters over the field (or at least that they are isotropic since they do not depend on the heading of the animal). Hence, we propose an extended version of the BW model in which the three parameters are allowed to depend on the orientation field or, equivalently, on the heading of the animal with respect to the global direction given by the inclination. This *extended Boltzmann Walker* model is presented in the section *Analysis*. It allows us to examine separately the effect of the inclination on the three parameters. We validate that these effects quantified at an individual scale yield back population statistics which are compatible with those observed.

Finally, we discuss how this extended Boltzmann Walker model can be used in contexts of more natural landscapes with heterogeneous inclinations.

## Results

### Data set

For experiments, a 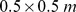 virgin painting canvas was set up under HD video camera recording (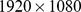 pixels), and tilted with various inclinations 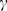 by 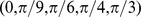 rad. Since we know that ant motion can be greatly affected by temperature [31], [32], the experiments were performed within a climatic room in order to control precisely the temperature (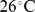) and relative humidity (50%). For each inclination and each of 3 colonies, 23 ants were collected from their housing container and placed within a Fluon-coated bowl, with a tuft of cotton soaked with sugar water. Then, each ant was gently picked up in turn using a small pig hair paintbrush, and the brush head was lowered to touch the canvas at the center point, where the ant could spontaneously walk down from the brush onto the canvas (see Movie S1 illustrating such an event). Ants could take up to seven minutes to walk down from the brush, but they usually made it in approximately one minute. This careful procedure ensured that the ants displayed a spontaneous behavior and not an escape response. Note that the ant *Lasius niger* is known for not displaying active trail-laying behavior in an exploratory context, and that the passage rate on the canvas excludes effects from passive pheromone deposition (area marking by footprint hydrocarbons laid passively by walking ants) [33]. The ants were then filmed until they exited from the canvas frame. From the 50 Hz interlaced video recordings, a custom tracking software extracted the position of the ant at each frame with sub-millimeter precision (see Movie S2 illustrating a short sequence of tracking). These tracked points were finally sub-sampled at 25 Hz. To avoid taking into account the very first moments of ants experiencing a new surface, we have discarded the early part of the trajectory up to the time when the ant had walked at least 1 cm away from its dropping site. To avoid the geometrical bias due to the square shape of the canvas, we defined the end of a trajectory as the point where the ant exited the 0.2 m radius circle centered on the starting position. Overall, we obtained 69 trajectories for each inclination 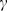, yielding a total of 345 trajectories, representing 845263 data points (Min = 127, Median = 1798, Max = 14434 data points per trajectory).

We believe that this experimental data set benefits from being well controlled for factors affecting the ants' motion (temperature, humidity, stress), and uses high tracking precision to determine ants' positions. As we do not claim that the modeling framework we use below is exhaustive by nature, we made the whole data set available as supplementary information so as to offer the community an opportunity to analyze the ants' trajectories from a complementary point of view (e.g. with potential field approaches [34], [35], continuous time analysis [36], [37]) or further analytical account of the observed directional persistence due to support inclinations (e.g. [38]).

### Effect of inclination on the time-averaged statistics of ants' motion

**Figure 1:**
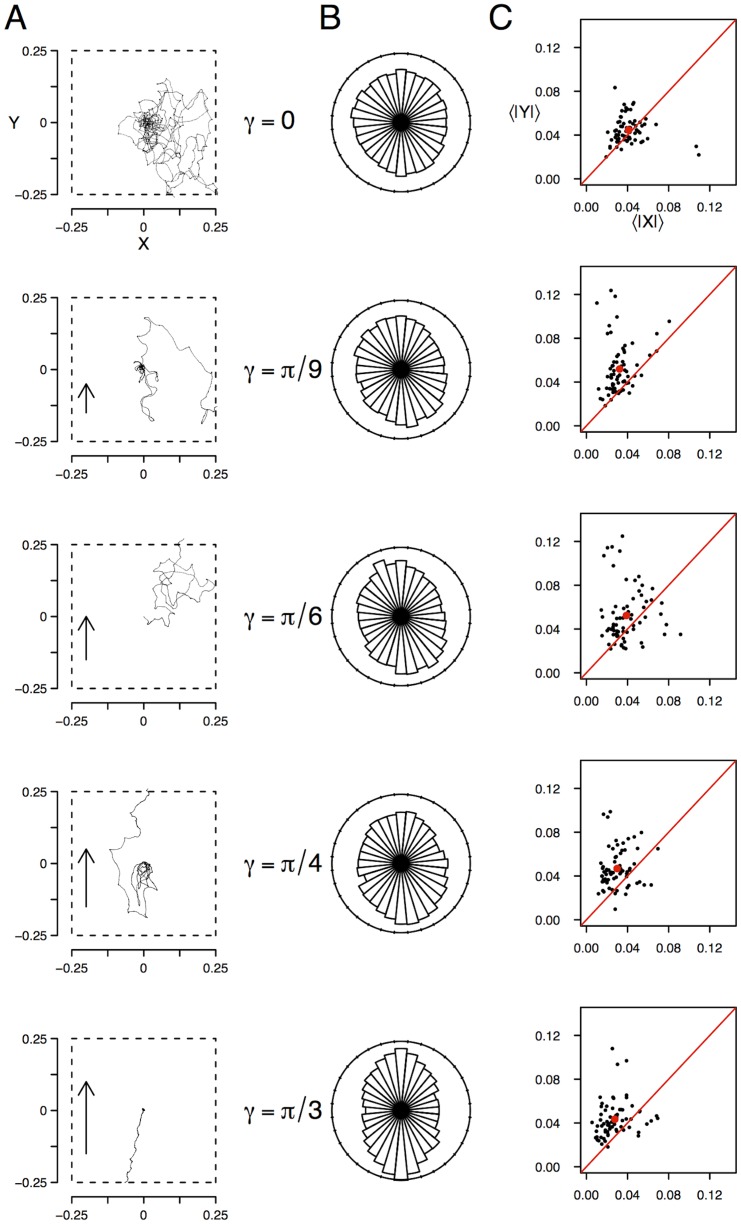
Effect of the support inclination on A — typical trajectories of ants, B — statistics of headings and C — statistics of positions. Slopes are indicated by labels 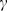, and illustrated by the (arbitrarily) increased length of the vectors on the left, heading uphill. Trajectories are 8.95, 2.28, 1.86, 2.30 and 0.67 meters long respectively. The statistics of headings, shown in B, compiles all ants' headings over time estimated every second. They show that ants are more and more often aligned with the steepest line as the inclination becomes steeper. Over time, this consistently biases the positions of ants towards locations uphill or downhill (up or down on the graphs A). This bias is summarized in C, using as proxies the absolute values of horizontal 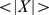 versus vertical 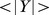 coordinates of ant locations averaged over time for each ant (one dot per ant) for each inclination (red dot: 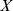 and 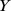 locations averaged over time and ants, red line: 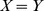). The higher values in 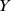 indicate that the ants are on average further away from the center along the steepest line (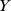 axis) than along the horizontal line (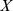 axis), meaning that ants are more dispersed in the 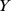 direction. Both types of distributions are significantly different from homogeneity even for the smallest incline 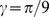.

Examples of ant trajectories on the inclines are illustrated in Fig. 1A. Typically, for higher inclination 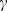, trajectories are more and more elongated along the line of maximum declivity gradient (hereafter, *steepest line*). The inclination-averaged time-averaged statistics of headings, shown in Fig. 1B, consider all ants' headings estimated every second. They confirm that ants were found more and more often aligned with the steepest line as the inclination was higher. A circular test, relevant for bimodal distributions [39], [40] shows that the distributions are significantly different from uniform even for the smallest inclination (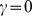 : 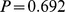; 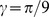 : 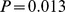; 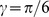 :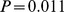; 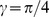 : 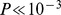; 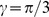 : 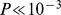). Over time, these orientation effects consistently bias the positions of ants towards locations uphill or downhill and translate into a change of space occupancy, as the ants spread more in the direction of the steepest line, namely more vertically than horizontally. To illustrate this effect, we have used the absolute values of horizontal versus vertical coordinates of ant positions averaged over time as proxies (Fig. 1C). The higher values along the steepest line are an indication that here the ants are found on average further away from the center along the steepest line than along the horizontal line, meaning that ants are more dispersed on the steepest line direction (Two-sample Kolmogorov-Smirnov testing the homogeneity of the distributions along the steepest line versus along horizontal line : 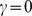 : 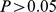; 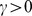 : 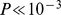).

**Figure 2:**
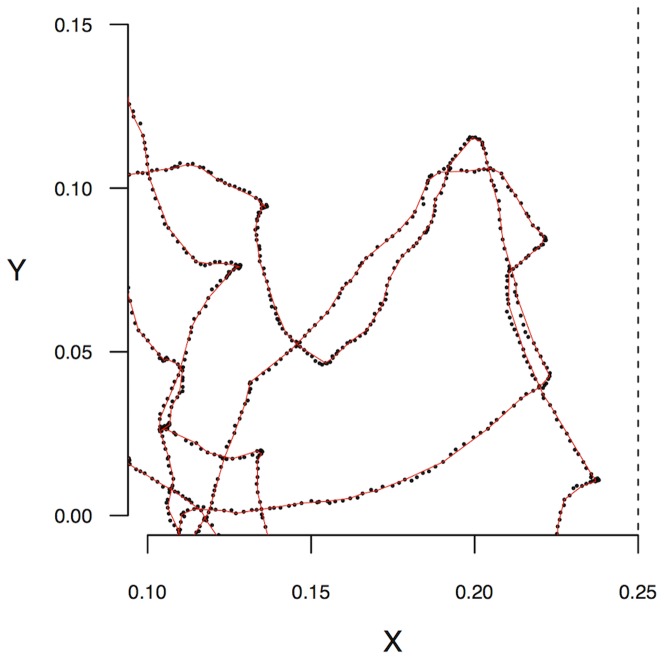
Typical example of a segmented trajectory. A portion of a trajectory is shown in dots (same ant as in Fig. 1A for the null inclination). The segments resulting from algorithm 1 are shown as red lines.

In a second step, using the noisy tracked positions, we recovered a representation of the ants' trajectories compatible with the Boltzmann Walker description. For this, the time series of detected locations were converted into a series of straight segments separated by reorientation events. A full description of this segmentation procedure is given in the Methods section, and a typical result is illustrated in Fig. 2. As a result, we obtained for each ant 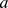 on inclination 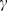 a series of 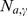 segments of various length 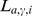 with headings 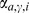 and 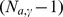 reorientations events quantified by the corresponding smallest signed deviations 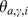 recovered following:
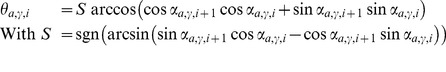
(1)


Overall, we obtained 345 trajectories (69 per inclination value) containing from 3 to 2246 segments. The numbers of segments per trajectory for each inclination were (min–median–max) 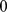 : 11–228–2246 ; 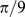 : 11–243–1816 ; 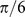 : 11–110–1334; 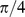 : 9–84–573 and 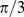 : 3–70–400. The total numbers of segments for each inclination were: 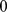 : 24456 ; 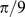 : 23801; 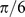 : 11663; 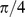 : 7318 and 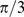 : 5985, with the total number of segments being 73223.

**Figure 3:**
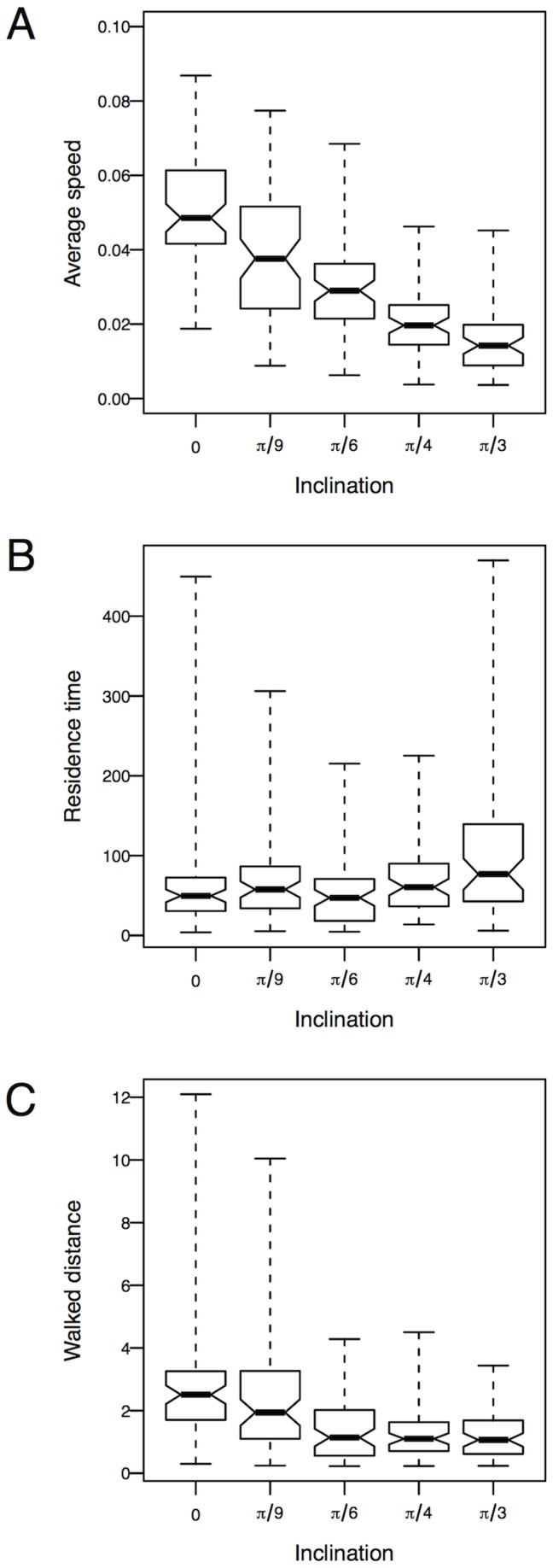
Effect of the support inclination on A — the average motion speed, B — the average residence time and C — the average trajectory lengths. These quantities are computed over 69 trajectories for each inclination. The inclination has a major impact on the motion speed, which in turn induces longer residence times. However, since ants move straighter towards the upper or lower edges when the inclination is steeper, their total trajectory length within the disks is lowered.

From these trajectories, we derived individual time-averaged statistics such as the time needed to reach the border of the outer circle of radius 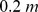, and the walked distance and the corresponding average motion speed within this area. The inclination was found to have a major effect on the motion speed of the ants; the steeper the inclination, the slower the ants (Fig. 3A, 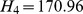, 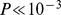). This lower speed consistently induced a longer time to reach the edge (Fig. 3B, 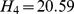, 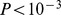). However, we observe that on the highest inclination ants display straighter trajectories, mostly aligned with the steepest line. As a consequence, their average trajectory length is approximately half as short as in the reference case (Fig. 3C, 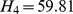, 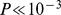).

Thus far, these time-averaged statistics confirm that inclination has a major effect on speed, but also that ants adapt locally their decision making about where to go, and/or how long to persist in the same direction, depending on how they are aligned with the steepest line. To give a full account of how the support inclination affects the ants' trajectories, we propose a behavioral model which accounts for this effect at the individual scale, as a stochastic decision process all along the trajectory. This model is developed by extending the standard BW model.

### Analysis of trajectories using the Extended BW model

#### The Extended Boltzmann Walker model

The classical Boltzmann Walker model is summarized in the Methods as a reference. In short, the BW model describes the behavior of diffusive walkers with two main components: straight paths separated by instantaneous reorientations events. In the purely diffusive version, homogeneous in space and time, and at constant speed, the memory-less nature of the process entails an exponential distribution of the lengths of the paths, with a characteristic length 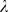 (or a corresponding spatial frequency 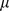, in 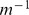). The reorientation events are governed by a probability density function 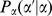 choosing a new direction 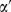, which is symmetric around the incoming direction 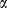, and can be more or less concentrated around it. For most forms of 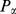 (e.g. elliptical), this concentration can be quantified by 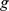, the mean cosine of the orientation deviation, which indicates the heading persistence (from 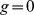 for a complete reorientation process, or null persistence, to 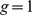 for null deviations, or complete persistence).

Being memory-less, the stochastic behavior of the Boltzmann Walker can furthermore be translated with no approximation into partial differential equations describing the time evolution of the probability density 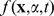 finding the walker at location 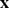, in the direction 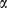 at time 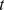. This yields the well-known Boltzmann equation (see Methods). When this model is used to describe linear transport systems in a homogeneous medium, e.g. photons scattering in a cloud, it is usually taken for granted that speed 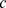, the mean free path 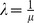 and phase function 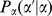 are independent of the incoming direction 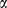. Moreover, external influences such as gravity (e.g. acting on molecules described as random walkers when analyzing gas diffusion) would be described by an additional term to account for forces.

In contrast, the effect of support inclination 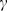 on an ant's decision making process will be studied by analyzing how those three parameters are affected by 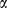 so that ants are found more often aligned with the steepest line, depending on inclination 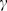 (we exclude a direct action of gravity, so the inclination effect is purely mediated by the behavioral decision).

Introducing the full dependencies of these parameters, the extended version requires:
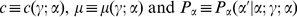
(2)There are three main predictions compatible with the higher probability of finding ants aligned with the steepest line:

- a – When ants are aligned with the steepest line, they become slower
- b – When ants are aligned with the steepest line, they increase their path lengths on average
- c – When ants take new directions, they favor uphill or downhill directions

The first two predictions are a type of ortho-kinesis and klino-kinesis respectively, the third being a kind of taxis. Note that we assumed here that speed fluctuations (among and/or within individuals) are governed by a process uncorrelated with the reorientation and persistence decisions, and remain to be studied separately, if relevant. Hence speed, the mean free path and phase function are treated in this context as independent parameters. Accordingly, in the next part, the three predictions will be tested independently, and for each inclination 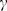 separately. For prediction (a), we will test whether the average speed depends on the current walking direction 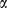. For predictions (b) and (c), we will test whether the geometrical properties of the trajectories (mean segment length, angular deviation between consecutive segments) also depend on direction 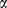.

#### Analysis

**Figure 4:**
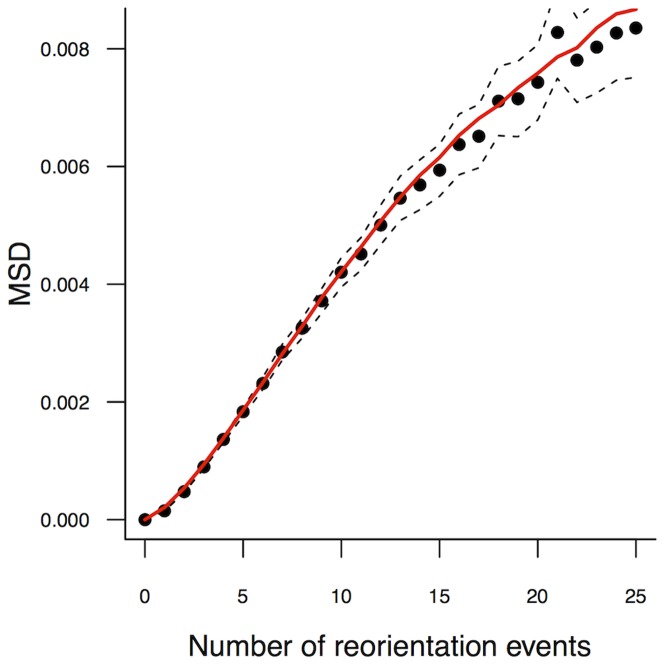
Mean Square Displacement of ants on the horizontal plane (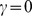). The MSD (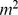) is shown as a function of the number of reorientation events along the trajectory. Points and dotted lines report the observed values (mean, 95% CI). The square-curve “ballistic” shape for few events are a trace of the direction persistence of ants, which disappears after some direction changes, yielding then a linear dependence of the MSD to the number of reorientation events, a well-known indication of diffusive behavior. For even larger numbers of reorientation events, the censoring effect of the domain frontier becomes dominant. The red line reports the MSD predicted by simulating the isotropic BW with the parameters estimated from the segmented trajectories for the null inclination.

First, using the Mean Square Displacement, we checked that *L. niger* displayed diffusive behavior in the horizontal condition of the present setup, as expected from previous studies. Since motion speed can vary among ants, even for the same inclination, we report it as a function of the number of reorientation events, which is insensitive to speed variations (Fig. 4). The observed pattern is clearly consistent with a diffusive motion at the statistical scale. This is a strong indication that the BW model is relevant and, importantly, it validates the segmentation procedure which yields the correct measures of the mean free path and phase function consistent with the observed dispersion rate.

**Figure 5:**
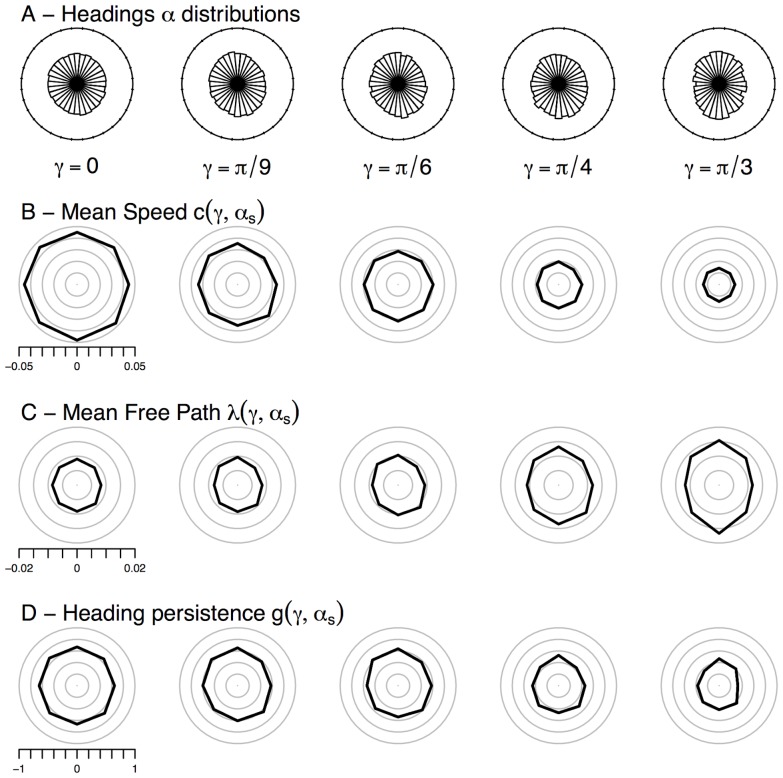
A — Distribution of headings 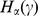, B — corresponding motion speed 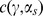, C — mean free path 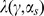, and D — 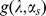, the asymmetry coefficient of the phase function. The heading domain has been split into 16 sectors (each centered on the corresponding 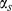). The upper row shows for inclinations 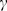 increasing from left to right the proportion of segments headings falling in each sector, and the lower rows show the average quantities computed over the corresponding segments sets. Std. err. for B, C, D are in the order twice the thickness of the line.

Then, using the segmented series, we computed for each inclination the frequency distributions 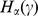 of the ants' headings 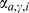. To examine the influence of the current heading 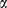, we split the set of segments into 8 heading sectors 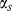, and computed the corresponding average speed 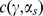, mean free path (average segment length) 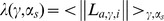, and heading persistence associated to the asymmetry coefficient of the phase function 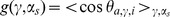, where 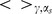 denotes averaging over sector 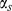 and inclination 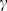. The results are shown in Fig. 5.

For the null inclination, the distribution of the headings is flat (Fig. 5A, 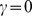), and the distributions of the speed, mean free path and heading persistence are all isotropic (Fig. 5B,C,D for 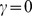), which confirms that the ants are well described by the isotropic BW model on the horizontal plane. As the inclination increases, the distributions of the headings are skewed towards directions aligned with the steepest line (Fig. 5A), in agreement with the distributions of 1 s-step sampling of headings shown in Fig. 1B.

Using the sector splitting of the parameters, we can now test each prediction in turn.

#### Prediction a – When ants are aligned with the steepest line, they become slower

Regarding the average speed 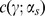 (Fig. 5B), beyond the global reduction found above (Fig. 3A), we found no indication that the average speed would be affected by the current alignment of the ant with the steepest line, for any support inclination.

#### Prediction b – When ants are aligned with the steepest line, they increase their path lengths on average

**Figure 6:**
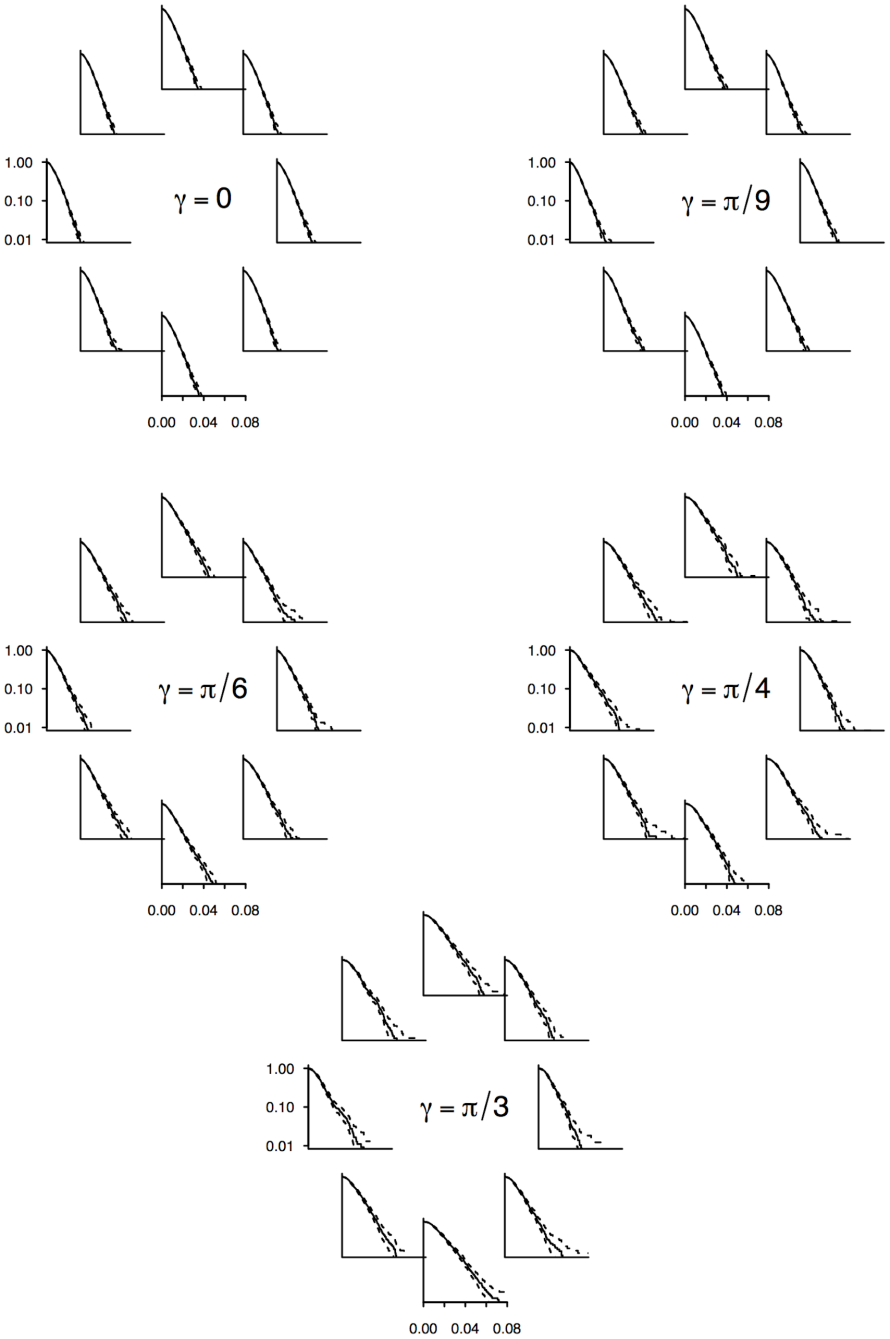
Segment length survival functions depending on the incoming direction and support inclination. The orientation domain has been split in 8 sectors. Segments were partitioned in sectors according to 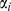, the incoming direction of the ant. Graphs represent the corresponding survival distributions of the segments' length. The corresponding phase functions are shown in Fig. 7.

We observe that the mean free path 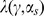 (Fig. 5C), remains the same magnitude on average for all inclinations. However, there is a strong indication that the angular distributions of 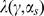 show a shift from the isotropic shape found for 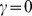 towards an anisotropic shape for steeper inclinations, with a skew in favor of segments aligned with the steepest line. This means that ants would walk longer when they are aligned with the steepest line (Figs. 5C and 6). Importantly, when segments are aligned with the steepest line, the increased length is almost the same either uphill or downhill, while it remains close to the value found for the null inclination when the ants are moving horizontally.

#### Prediction c – When ants take new directions, they favor uphill or downhill directions

**Figure 7:**
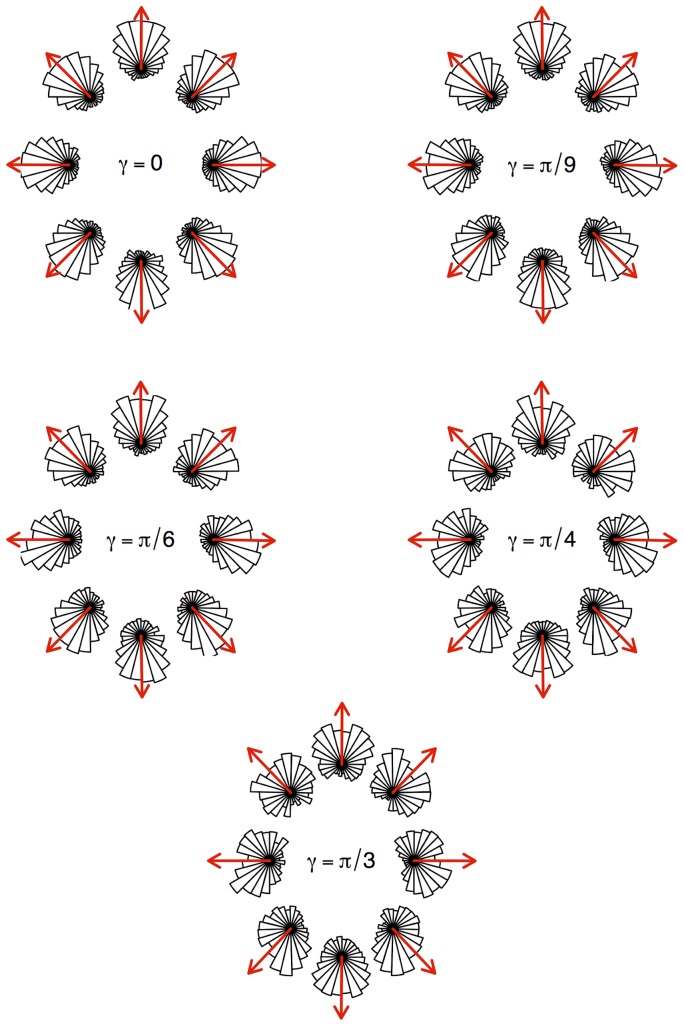
Phase function depending on the incoming direction and support inclination. The orientation domain has been split into 8 sectors. Segments were partitioned in sectors according to 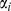, the incoming direction of the ant. Graphs represent the corresponding distributions of the next direction deviation 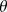. Red arrows are the average 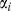 in each sector.

The concentration of directional deviations, or heading persistence 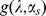 also seem to be affected by the inclination (Fig. 5D) with ants losing some persistence as the inclination is steeper. In addition they seem to be affected very little by the current walking direction 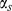. However, we found that the very shape of the phase function was actually affected by the incoming direction 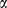, we therefore reported the phase function separately for each sector (Fig. 7).

We found a major effect of 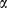 such that the shape of the phase function appeared to depend both on the inclination and the incoming direction. Obviously, and here again in accordance with the isotropic BW model, the phase function is the same for every incoming direction in the case of the null inclination. However it appears more and more skewed towards uphill and downhill directions as the inclination increases. Significantly, the phase functions still display mirror symmetry for pairs of opposite incoming directions (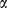, 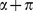), albeit with different shapes when heading horizontally (right/left) or vertically (up/down). When the incoming direction is vertical, either up or down, the ants tend to persist in their direction in the same way as when they walk on the null inclination. When the incoming direction is horizontal, the phase function becomes less concentrated to small deviations, especially for the steeper inclination for which it becomes poorly persistent: at reorientation events, ants tend to depart directly from the horizontal line in either uphill or downhill directions, with a likely preference towards downhill directions. For intermediate incoming directions, the phase function becomes even more asymmetric, with a higher concentration when turning towards the closest vertical heading, especially when it is downhill, and is less concentrated otherwise.

**Figure 8:**
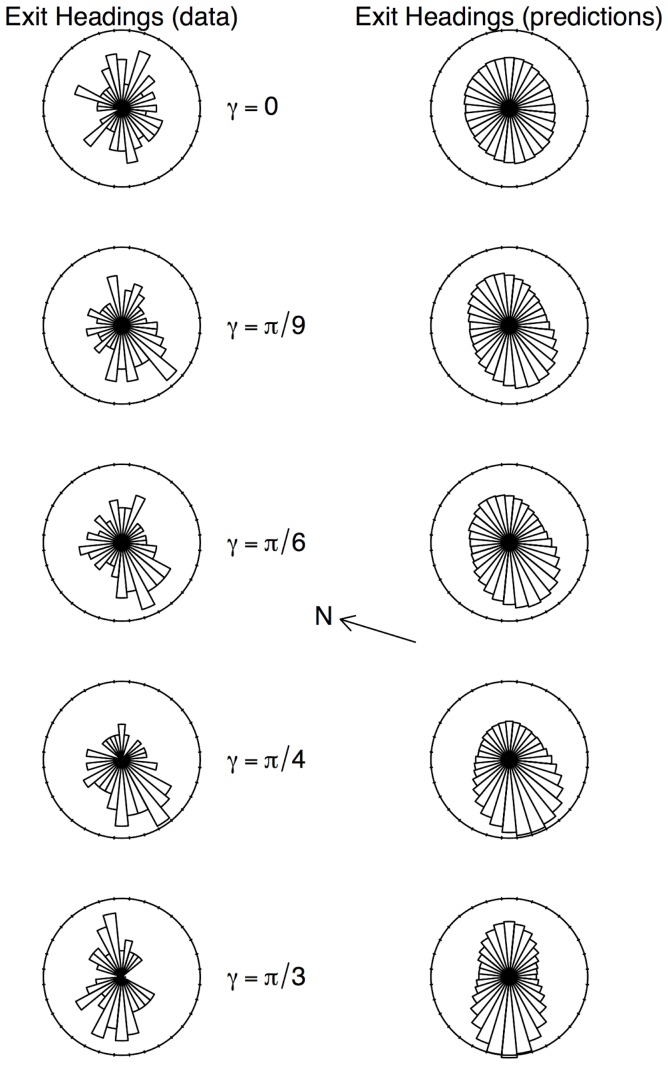
Observed (left) and predicted (right) statistics of the exit heading, for each inclination. The exit headings were computed for each ant as the direction from the starting point to the point where they exited the disk of radius 0.2(left, N = 69 per 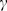 value). The arrow indicates the direction of the magnetic North (N). Simulations of trajectories were performed using the sector-based statistics of the phase function and mean free path (N = 100,000 simulations by inclination, see *Methods* for the simulation algorithm). The corresponding predicted exit headings distributions are shown on the right. The compatibility of the data with the predictions was tested using the standard Kolmogorov-Smirnov test for the null hypothesis that the two distributions were drawn from the same continuous distribution. This null hypothesis is not rejected for any inclination. The consistency of the data and the predicted distributions indicates that the impact of the inclination on the ant motion is comprehensively captured by the induced change in the BW model features.

As a final check that these observed effects of support inclination on the extended BW model features and parameters are fairly consistent with the observations at the population scale, we have generated numerically trajectories using the parameterized model (see Methods). We report the observed and predicted distributions of exit headings in Fig. 8. The predictions recover well the general trends of the ants' statistics, as they capture both the higher probability to exit uphill or downhill, and also the downhill exit preference.

Overall, this analysis shows that the extended BW model parameters undergo two kinds of effects as the inclination increases, and the two predictions (b) and (c) should be considered. As for the prediction of klino-kinesis (b), ants moving on the steeper inclination actually appeared to increase their path lengths, on average, when they are aligned with the steepest line (Fig. 5C). As for the choice of the new direction at the ends of their free paths (taxis, c), they also modified their choice when their incoming direction was horizontal with a marked preference for choosing new vertical headings. For intermediate incoming directions, ants favored up or down directions depending on which is closer. Consistently, they also appeared to maintain more often their current heading when they were already walking uphill or downhill.

## Discussion

In this study, we have performed a detailed analysis of how *L. niger* ants move on an inclined support, examining even at the scale of their decision making processes. We have first revealed population level statistics which show indeed that the support inclination affects the ants' dispersal, and we propose in the end a behavioral model of their random walk that embeds the influence of this inclination on their decision about where to move to, and for how long: the *extended Boltzmann Walker* model. The standard Boltzmann Walker model is a model of reference to describe the random walk of ants on a horizontal plane. It was extended to incorporate the different possible effects the support inclination could have on the decision making of walking ants. The extended model was parameterized using a high-quality set of data, and the measured functional dependencies of its parameters on the inclination show how the latter affects these decisions. At this individual level, we found that the directional field given by support inclination affects ants' motion decisions on two parameters, depending on their incoming direction (or alignment with the steepest line): the mean distance between turning events (mean free path) and the choice of the new direction at turning points. Thus the behavioral decision process appears to combine klinokinesis with taxis. We also found that the support inclination had a major impact on the average moving speed of the ants, but this effect was isotropic and did not depend on the incoming direction. The model establishes the correspondence between the individual stochastic motion decisions governing the random walk process and the prediction of the anisotropic dispersal of the population (Fig. 8).

The present set-up was designed to isolate the effect of inclination on the ants' decision-making, so as to identify and quantify this effect. To this end, we managed to maintain the inclination the same all over the field, and keep everything else as constant as possible. In this homogeneous field, we consistently assumed that the influence of inclination on motion decisions was the same everywhere. It is noteworthy in this case that the lower speed on steeper inclines is more or less compensated for by straighter trajectories, so that the mean residence time in a definite area is only mildly affected by inclination. Further theoretical developments are required to derive the macroscopic equations corresponding to the extended BW model in the case of such a homogeneous directional field. Such a derivation of invariant characteristics (oriented diffusion, residence time, first-return statistics, statistics of visits…) is however expected to be challenging, especially considering the asymmetric shape of the phase functions for intermediate directions (Fig. 7), which precludes the reciprocity of paths, a standard requirement for the diffusion approximation. In fact, in the present state, it is likely that such macroscopic features are to be derived numerically in most cases.

Moreover, the most interesting biological situations arise naturally for landscapes of varying inclinations. Since the characteristic shape of these variations (e.g. spatial frequencies spectrum) will probably be case-specific (dispersal within the nest, foraging in the external environment around the nest entrance, migration, etc), the functional consequences of the reaction to support inclination is expected to be highly context-dependent. In the context of building behavior, the next step will be to establish how the distributions of visits inside a given structure is affected by the preference for alignment with the steepest line (versus a uniform distribution predicted by pure diffusion).

The extended Boltzmann Walker model is a time-continuous description of the motion built upon the assumption of a memory-less process, so decision-making is considered instantaneous at the model time-scale, and only depends at any time on the information perceived at position 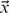 at that time. So it remains fully appropriate in the context of a varying inclination, as the extension (2) simply translates to:
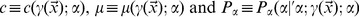
(3)


The predictions about how the extended BW model would shape the distribution of ants in a given landscape call for dedicated numerical studies, using Monte-Carlo simulations in complex geometries. There is no additional need for simulating the choice of a new direction since it remains a purely local decision at turning points. However, it would require specific algorithms (such as a null-collision algorithms [41]) to cope with heterogeneous distributions of the mean free path, and computation with no approximation of the locations where the individual makes heading turns. Such an algorithm will need to be further adapted to also take into account the effect of inclination on the average motion speed.

As for the speed variance (either for one individual across time, or among individuals), we have indicated that we focused on the geometrical aspects of the trajectories, considering the speed process as independent. As a matter of fact, this assumption is well supported a posteriori by the result that we did not find an effect of the heading on the average speed of ants, that is, the speed process does not seem to be affected by directional information. As it is known that speed can vary with temperature, replicating the same study with higher and lower temperatures would constitute a good test for the independence between the process governing speed, and the two processes governing trajectory geometry, which we have assumed here.

Considering macroscopic statistics, using average speed and neglecting speed distribution has proven to be a fair approximation in previous studies [1], [6], [13]–[17]. Accordingly, we suggest using average speed as a first order approximation, as long as typical speed variations are the same scale as the mean free path. Under this condition, speed fluctuations would impact only mildly on measures averaged over large time scales. If this approximation is to be rejected based on experimental grounds (e.g. caste-dependent average speed, or daily-scale variations), it might become necessary to investigate more closely the impact of speed distribution on average statistics, depending on the biological question at hand.

### Open questions

The isotropic distribution of average speed appears as a surprising result since it would be expected, for instance, that ants progressing uphill should be slower than when moving downhill. For instance, Seidl et al. found lower speeds on steeper inclines in desert ants moving uphill, but indicate that desert ants progressing downhill displayed high velocities [42]. This is in contrast with our finding with *Lasius niger* in the present set-up where the velocity showed no dependency to the walking direction, even for the steepest inclination. However, Wohlgemuth et al. report, also in the desert ant, that speed was reduced in both their uphill and downhill channels (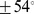) compared to their flat channel, thereby excluding metabolic cost as a reliable means to gauge walked distance on various inclines [28]. In an attempt to determine the effects of inclination on the gross metabolic cost of locomotion in leaf-cutter ants, Holt & Askew report that ants travelled the fastest on a horizontal plane, and indeed moderated their speed with the inclination, both on the incline and the decline. They suggest that ants adapt their behavior so as to keep their metabolic rate constant despite changing mechanical demands [43]. The issue of energy demand and climbing behavior in small animals was raised by Full & Tullis [44], who pointed out that this demand should be minimal. Consistently, Lipp et al. refute an energy-based mechanism for slope angle measurements in small ants such as Camponotus because the relative cost of vertical locomotion should be smaller in smaller animals, and become negligible with regards to the relatively larger cost of basic metabolism required for just walking [45]. These contrasting findings in different species may of course stem from the species *per se* (e.g. the inclination has no effect on speed in the wood ants [42]), but also from the different behavioral tasks the ants had to face and the different experimental situations in which those measures were carried out. For instance, in the context of following foraging trails on inclines, leaf-cutter ants show a behavioral plasticity in selecting their load size, likely because the inclination had a significant effect on their walking speed [46].

Regarding the statistics of exit heading direction (Fig. 8), we observed a visible excess in favor of the lower right part of the canvas, which is mostly present for intermediate support inclinations (and disappears for the steepest one, 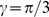). We have no explanation for this bias so far, and it calls for further examination and testing. For instance, eusocial insects are sensitive to the magnetic field [47]. Sandoval et al. have shown recently that *Solenopsis* ant orientations are affected by changes in the magnetic field direction in a context of exploration (on the level plane) [48], and *Atta colombica* were also shown to use magnetic information for their path integration [49]. We have indicated the direction of the magnetic North on figure 8, but the skew observed in the exit headings does not align with it, and we are not aware of studies about magneto-reception in *Lasius niger*.

More generally, the coarseness of the substrate on which ants are moving should also be considered, as it can impact greatly on both speed and the sinuosity of trajectories [17]. In addition it is likely that ants' motion behavior should be affected by physical parameters such as temperature, wind or even air humidity. In principle, the extended BW model would allow the incorporation of any combination of these effects within the same framework, and the experimental task would consist of revealing how the three extended parameters are affected by each kind of information. In the spirit of Weitz et al. [6], we advocate that this framework is then a good starting point to design the relevant experimental setups. For instance, ants might also adapt their paths according to the local curvature of the support, in addition to its inclination. Since curvature is indeed a spatial variation of inclination, both effects can be difficult to disentangle. The extended BW framework could help in designing the most efficient experimental measures that should be taken to discriminate between both effects.

Finally, is this influence of ground inclination relevant for contexts other than ants' motion behavior? Understanding how animal movements are explicitly driven by environmental factors is a challenge for further advances in dispersal ecology [50]–[52]. One major constituent of landscapes are spatial variations in declivities and hills. The *extended Bolztmann Walker* framework and the methodology presented above for using it in quantification and prediction of animal movements might be of help in such studies.

## Methods

### Ants collection and housing

Three colonies of ants *Lasius niger* were collected along the south part of the river Garonne, about 30 km south of Toulouse, France, on a private property with the permission of the owner. *Lasius niger* is not a protected nor endangered species. Our experiments complied with the laws and ethical recommendations currently in effect in France where the experiments were performed. Colonies were housed in plastic containers, and fed ad libitum with sugar water and Bhatkar preparation [53]. The experiments took place in a facility provided by the Ecole des Mines, Albi, France (E 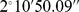, N 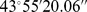). The upper end of the steepest line of the canvas was heading ENE (Grid azimuth = 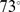, the magnetic North is indicated on figure 8, the magnetic declination of the site is approximately 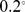). The timetable of the experiments is given in Table 1.

**Table 1:** Experiments Timetable.

Inclination	Colony	Day (YYYY-MM-DD)	Hour (HH:MM–HH:MM)
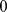	D	2012-05-29	14:15–15:00
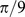	D	2012-05-29	15:25–16:20
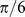	D	2012-05-29	16:25–17:30
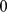	C	2012-05-31	15:45–16:40
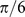	C	2012-05-31	17:15–18:05
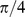	C	2012-06-01	11:20–12:35
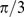	C	2012-06-01	16:55–18:45
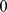	C	2012-06-01	19:45–20:50
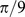	C	2012-06-04	11:30–12:40
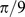	C	2012-06-04	14:10–14:40
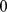	A	2012-06-15	14:00–15:45
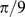	A	2012-06-15	16:10–17:30
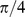	A	2012-06-15	17:50–18:40
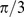	A	2012-06-18	11:30–12:50
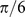	A	2012-06-18	14:50–15:30
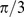	D	2012-06-18	16:00–17:20
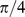	D	2012-06-18	17:40–18:50

### Computer tracking procedure

The tracking program was written from scratch using the Core Image infrastructure of Mac OSX (Objective-C+GPU-based Image manipulation), starting from the CIColorTracking example ([54]). Each movie frame was successively applied with the filters CIGammaAdjust (with inputPower 0.3), CICrop (with inputRectangle set as a 40×40 pixels square centered around the latest detected location), CIColorControls (with inputContrast as 3.5), and the CIColorTracking *ad hoc* filter MaskFromColor (with inputThreshold 0.27083 and inputColor defined by the user clicking on the background color in the first frame). This yielded a binary representation of the 40×40 pixels area containing ON-pixels corresponding to the ant and noisy speckle from background, from which the centroid of the largest spot was computed, using a partition algorithm where two adjacent ON-pixels were considered to belong to the same spot. A short recording of a typical session is given as supplementary Movie S2.

### Data availability

The whole set of data is made available as supplementary information. The data are given as supplemental data files (zip archives) : 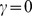 : Dataset S1; 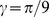 : Dataset S2 ; 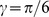 : Dataset S3 ; 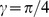 : Dataset S4 ; 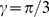 : Dataset S5.

Each archive file contains a series of 69 files, one file per ant. Each file contains the data of a trajectory in a tab-delimited text format with 9 columns, corresponding in order to the inclination index, the colony index, the temperature, the humidity, the recording date, the individual index, the rank of the video frame, the corresponding time in second, and the x and y coordinates in meters. Each file starts with a header line labeling this information.

### Estimates of heading distribution from raw data

For the distribution of headings over the time shown in Fig. 1, each trajectory was split into a sequence of 1-s periods, corresponding each from about 20 to 50 data points, depending on the speed. The local orientation of the trajectory was computed as the orientation of the axis corresponding to the first principal component of the cloud of points, using the R function *princomp* [55]. The circular histogram of the values were finally computed using the function *rose.diag* of the R package *circular* [56].

### Segmenting trajectories into sequences of straight free paths

We will detail in this section the algorithm we used to split the ants' trajectories into series of consecutive segments. Our algorithm is sourced from the field of time series data mining. This matter has received much attention over the last decade in relation with the increase of computer power and the explosion of data time series in a wide range of fields, from Life Sciences [57] to Telecom [58] and Image Processing [59]. The so-called piecewise linear approximation of a temporal signal is widely used to support clustering [60], classification and context recognition [58], [61]. Three major segmentation approaches can be distinguished: the sliding window, the top-down and the bottom-up algorithms. An extensive comparison between these approaches is given by Keogh et al [62]. The first is the most intuitive approach but gives the worst result [63]. Both latter ones operate on the whole set of points and the bottom-up approach is clearly the most reliable one.

The piecewise linear approximation in our context addresses the following problem: given a time series of locations in the plane, finding the best partitioning in linear segments. Such a process will thus aggregate consecutive points that belong to the same segment into one representation of this segment even if those points are not perfectly aligned. As an approximation, it can give a compact representation of the data, but compromises accuracy.

Hence the major concern for series segmentation is the balance between compactness and accuracy, i.e. the optimal number of segments [57], [64]. For a given series, the compactness can be evaluated by the number K of segments, and the accuracy should be evaluated by a distance between original data and approximation. In the words of Keogh [62], the balance criterion can be considered in several ways:

1. Given a time series T, produce the best representation using a fixed number 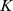 of segments.
2. Given a time series T, produce the best representation such that the maximum error for any segment does not exceed some user-specified threshold (local error, 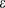).
3. Given a time series T, produce the best representation such that the combined error of all segments is less than some user-specified threshold (total error 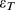).

The problem of finding the best partitioning is combinatorially complex, and the data time series are up to approximately 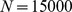 points long. We therefore designed a heuristic-based 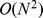 algorithm inspired from gradient-descent to derive the segments series from the points series. This algorithm is presented in Algorithm 1, and a typical result is shown in Fig. 2. We have chosen to follow the second criterion, and had to set a distance 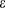, meaning that any original data point is not further than 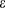 from the line segment it has been aggregated to. For a given time sampling of the ants' motion, the appropriate value of 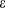 depends on the noise introduced by the tracking program: if the criterion is too low, the process of aggregating points into sets corresponding to segments stops too early, and lots of segments actually correspond to noise. Conversely, if the criterion is set too high, points are aggregated in too large sets, and we miss the details of the turning events. Hence, the confidence in this procedure ultimately calls for a fair estimate of the noise.

As a first check for the algorithm consistency, we have tested its performance on an artificial set of data in a zero-noise situation. For this, we have generated an artificial trajectory following the Boltzmann Walker model on a large area (1.5 m), with parameters close to the ones found in ants in first approximation: 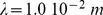, 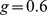. This trajectory is a sequence of 2266 segments separated by reorientation events, which have been sampled according to the elliptical sampling presented below in section Elliptical heading deviation sampling. This sequence was then resampled every 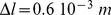 corresponding to an ant travelling at 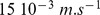 sampled at 25 Hz. This yielded a series of 37876 locations, given as the input to the segmentation algorithm, run with a very demanding criterion 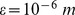. The output of the algorithm was 2037 segments, with an estimated mean free path 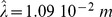 and an estimated persistence 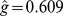. The missed segments correspond to very small angular deviations: two almost perfectly aligned segments are combined into one segment by the algorithm when their angular difference falls below the minimal angle associated with 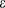. Both distributions of segments lengths and angular deviations were also well recovered (Fig. S1).

The next step was to estimate the sensitivity of the segmentation procedure to the accuracy of our estimation of tracking noise 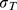. For this purpose, we carried out a cross-exploration of couples 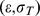, with 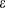 values in
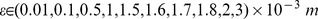
and 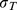 values in
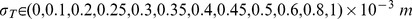



For each couple of values, we generated 300 artificial trajectories as above, using parameters 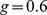, and 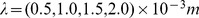, and retrieved the estimates 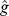 and 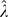. The results are shown in Fig. S2. Essentially, fixing our estimate of the tracking noise to 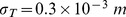, there exists a criterion 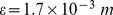 (Fig. S2, red lines) around which the segmentation procedure returns a fair estimation of both 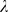 and 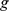, and more importantly in the present context, captures almost perfectly the varying 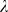. If we fix the criterion to this value, and vary the noise, the results appear also robust against a rough estimation of the tracking noise. Finally, since the criterion is ultimately a minimal angle of deviation between two consecutive segments, 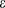 also depends on the spatial frequency of the data points, namely the mean distance covered between two consecutive points. With a sampling time frequency fixed by the video tracking, this implies that it depends in turn on average speed, so we scaled this criterion as speed decreases for steeper inclinations, following 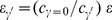.

**Algorithm 1** Piecewise linear segmentation of the trajectories.

The procedure is parameterized by a stopping criterion 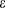, a distance.


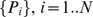
 are 2D-locations sampled at a constant time sampling rate.


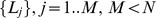
 are sets of consecutive 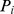 of various lengths.

Each set is associated with 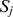 the segment delineated by the orthogonal projection of the two end points onto the major axis of the points cloud.

We denote 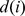 the distance from 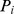 to segment 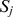 it belongs to.

We denote 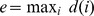 the error associated with 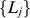.

We denote 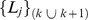 the segments series derived from 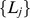 by merging the successive segments 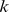 and 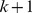, resulting in one segment extending from segment 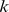 starting point to segment 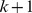 ending point. The derived series is one segment shorter than the original.

We denote 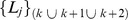 the segments series derived from 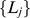 by merging the successive segments 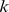, 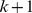 and 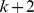, at the point 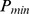 which introduces the minimal error, resulting in two segments extending from segment 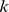 starting point to 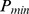, and from 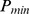 to segment 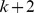 ending point. The derived series is one segment shorter than the original.

1: 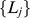 is initialized with the complete series of the shortest segments, joining every couple of successive locations: 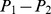, 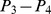,…, 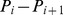


2: 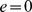 since all 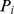 are endpoints of their respective segment.

3. **while** 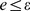 **do**

4:   **for each** 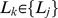 **do**

5:     Compute 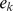 for 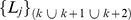


6: **end for**

7: 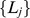 becomes 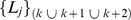 for which 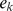 is minimal.

8: **for each** 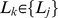 **do**

9:   Compute 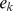 for 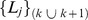


10: **end for**

11:  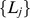 becomes 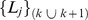 for which 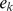 is minimal.

12: 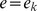


13: **end while**

### Mean Square Displacement computation

Since ants displayed varied speeds, and showed some periods of stopping from time to time, we computed the Mean Square Displacement as a function of the number of reorientation events rather than time, following [65]. For this, we used the trajectory representations given by the segmentation procedure. For each number of reorientation events 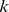, 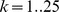, the trajectory was split into a sequence of 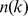 successive reorientation locations 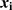 separated by 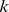 events. The MSD was then computed as:
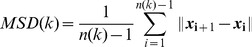
(4)


### Circular Statistics

In order to conduct heading statistics analysis, we used circular statistics, taking heading distribution as the input. Linear statistical measures cannot be used because angles on a unit circle have modulus 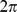 (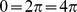 etc), and the fact that 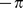 and 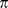 correspond to the same direction [66]. Given the shape of the distribution (with a combined skew towards upward and downward directions), we used a test for uniformity that is capable of dealing with bimodal data: the Hodges-Ajne test. This test is reputed to work well for bimodal or multimodal distributions. It was written with R [55] using code written for a MATLAB toolbox providing a useful approximation for large data sets, allowing us to avoid factorial calculations [39], [40]. The null hypothesis is that the population is uniformly distributed (isotropic). We can therefore compute the orientation direction when the null hypothesis is rejected. Since the distributions seem to be bimodal with two opposite modes, undirected axes have been computed. Following Batschelet [66], we double the angles and reduce them modulo 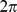 to obtain a unimodal circular sample. Let 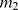 denote the mean vector with 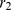 and 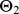 its polar coordinates. Let 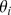 be one of the 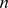 observed angles. Let 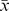 and 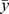 be the rectangular coordinates of the centre of mass of points projected on the unit circle. Then
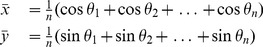
(5)

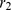
 is the mean vector length with components 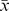 and 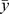:
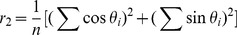
(6)


The mean angle 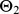 is obtained:
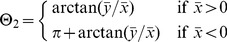
(7)


In order to obtain of the undirected axis angle of the original sample, we must cancel the effect of doubling:
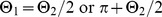
(8)

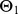
 defines the mean axis. In order to measure the dispersion, Batschelet proposes the mean angular deviation:
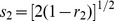
(9)


The final angular deviation value of our bimodal samples is:
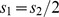
(10)


The function to compute the P-value for the test of uniformity was adapted for R from *circ_otest* of Circular Statistics MATLAB toolbox [39], [67].

### Simulating the extended BW model

When the need is to simply generate trajectories from the standard BW model, parameterized by a mean free path 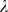 and a phase function given by an elliptical function characterized by the mean cosine 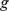, the numerical resolution can be done exactly, with no spatial approximation nor time discretization, following the algorithm 2.

**Algorithm 2** Generation of a standard BW trajectory.

Input parameters: mean free path 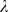, elliptical phase function of parameter 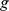


Variables: position 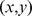, heading 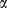.


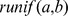
 returns uniform sampling in 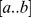



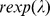
 returns exponential sampling of mean 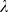.


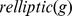
 returns elliptical sampling according to algorithm 4 below.


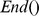
 is any condition to stop.

1: 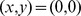


2: 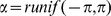


3: 
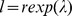


4: **while** (not 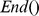) **do**

5:   
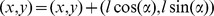


6:   
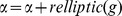


7:  
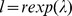


8: **end while**

To simulate the extended BW model, we need to further take into account the dependence of the parameters on the heading 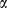. To this end, we have used the eight sector-based experimental distributions of mean free path and heading deviations shown in Fig. 6 and 7. Since we used eight sectors 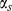, the resolution of the extended BW model is an approximation regarding angular dependence, but otherwise keeps the structure of the algorithm 2.

These simulations were performed in the R environment [55]. In order to program the random sampling function for each sector, and each parameter, the empirical cumulative distribution was first estimated from the corresponding data set, using 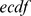. Then, this estimated function was sampled over an abscissa interval discretized in 100 bins, using 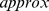.

For instance, let's denote F(a) as the discretized cumulated function of heading deviations for a given sector and a given inclination, with 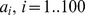 spanning 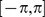. By construction 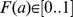, 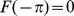 and 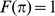. To draw random numbers according to 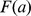, a uniform 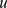 is drawn in 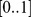, the lowest discrete abscissa 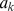 for which 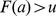 is found using 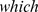, and the output value 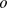 is computed by linear interpolation between this discrete abscissa 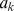 and the previous 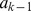, proportionally to their corresponding F values, namely:
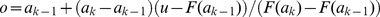
(11)


Denoting sector-based random sampling 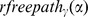 and 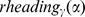 for free paths and turning angles respectively, generation of one simulated trajectory according to the extended BW model for a given inclination 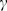 is given by the algorithm 3. To generate the predictions of exit headings, series of 10,000 trajectories for each inclination were generated following this algorithm, each starting from the center, and up to the exit from the 0.2 m radius of the circle (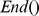 condition). Then the intersection point between the trajectory and the circle was retrieved, and its heading computed.

**Algorithm 3** Generation of an extended BW trajectory.

Input parameters: inclination 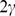, free path sampling function 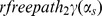, turning angle sampling function 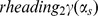



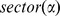
 returns the sector of 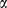.


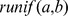
 returns uniform sampling in 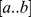


Variables: position 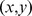, heading 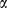.


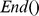
 is first exit from the 0.2 m radius circle.

1: 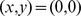


2: 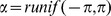


3: 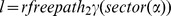


4: **while** (not 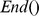) **do**

5: 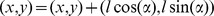


6: 
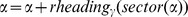


7: 
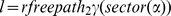


8: **end while**

### Elliptical heading deviation sampling

Generating artificial data required sampling the angular deviation according to a probability density function governed by parameter 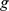, the average cosine of the deviation. We used an elliptical shape for this function. A random deviation can be drawn following the algorithm 4.

**Algorithm 4** Elliptical angular sampling.

Input parameter: 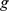.


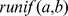
 return uniform sampling in 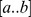.

1: 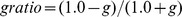


2: 
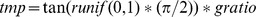


3: 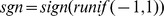


4: 
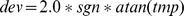


5: return 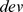


### The Boltzmann Walker (BW) model

In the *Boltzmann Walker* model, the particle or the animal keeps moving on a straight line until it punctually and instantaneously changes its velocity (orientation). Its path can thus be split into a sequence of linear segments. This model, inspired by the scattering behavior displayed by photons in participating media, has been called *Velocity-Jump process* in other fields [7], [8].

When particles such as photons are involved, velocity changes are triggered by local interactions with molecules or particles. As far as ants or other animals are concerned, the velocity changes *look* random, with no apparent events such as collisions, and the attempt to disclose the deterministic triggering mechanism (internal neural process, reaction to randomly dispersed indiscernible heterogeneities, etc) would be challenging in most cases.

However, this random component of the path can be precisely specified as follows: the velocity change can occur at any time, it does not depend on how long the animal has been walking since the last velocity change event — this is a memory-less process. Let 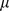 denote the rate at which velocity changes occur; the unit is the inverse of a distance, meaning that (in case of 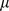 constant) an ant displays a velocity change every 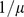 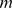 on average. It is worth noting that this quantity may vary in space and time under the leverage of environmental clues provided that this influence can be considered as instantaneous at the model time scale; thereafter, we shall restrict the analysis to specific cases where it only depends on the position (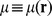).

Starting from the location of the last change, the probability that the next change does not occur before the ant has walked 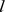 is thus given by :
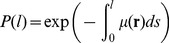
(12)with integration following the curvilinear abscissa along the trajectory.

If the rate is constant over space, 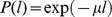 and the probability to carry on over 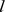 with the same velocity is indeed the survival function. We will denote the average distance covered between changes 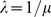 (in 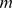), which is known as *mean free path* in statistical physics.

What happens at turning points under this model ? Let us denote 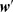 and 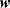 the unit direction vectors of two consecutive segments. The normalized distribution of direction changes 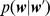 is also known as the phase function (or scattering indicatrix) in statistical physics. The quantity 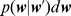 determines, for a turning event, the probability that an animal walking in the direction 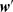, will be scattered within the limits of the elementary angle 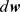 in the direction 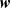. The normalization constraint is then:
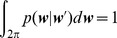
In the field of biology, this random walk is called a correlated random walk (CRW): the new direction is chosen with a particular shape of the probability density function according to the previous direction. It is common to observe in a social insect that forward scattering is dominant, meaning that the animal has a tendency to make small deviations at each reorientation. The particular case where 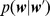 is uniform and indeed independent of 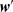 is named a pure random walk (RW). To characterize reorientation events with a single and scalar quantity, it is usual to define the anisotropy coefficient of the angular phase function 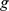 computed by:
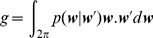
(13)

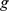
 collapses to 0 for a uniform phase function and tends to 1 as the deviations become smaller and smaller around the previous direction. It may also tend to −1 when the animal exhibits a strong propensity to take frequent u-turns.

It can be shown that the statistics of space occupancy corresponding to this model are well approximated by a diffusion process (see below). Moreover, the corresponding diffusion coefficient that would govern the spreading rate of a population over time is strictly related to the parameters of the individual decision model following (in 2D):
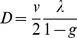
(14)so that, at the macroscopic level, the diffusion coefficient truly depends on the combination of the mean free path *and* the distribution of turning angles. Hence a macroscopic formulation of a *correlated random walk* driven by 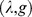 could be as well rendered by a *pure random walk* driven by 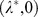 provided the mean free path 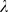 is tuned accordingly so that: 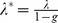. Statistical physics calls 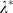 the *transport mean free path*.

#### Translation into a transport equation

With the BW model, a single walker is followed over time along its trajectory, making free paths and turning events. There is an alternative description focusing on what happens at a given position and a given direction over time. Let 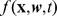 be the probability density the walker is at location 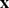 and walking in direction 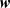 at time 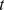 (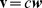 is the walker speed vector). The individual-based scale description of the Boltzmann walker can then be strictly translated into the following mesoscopic equation [13], [69]:
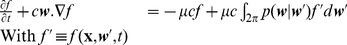
(15)


Equation 15 is a version of the well-known Boltzmann equation, when it is used for describing linear transport systems (e.g. photons scattering in a cloud).

Equation 15 can be integrated over directions to derive the evolution of 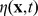, the density field of a population of BW (or equivalently the probability density field of a unique walker), yielding:
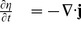
(16)where 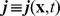 is the current density.

In the same way, Equation 15 multiplied by 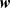 can be integrated over directions to derive an evolution equation for 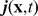. However at this stage, it is necessary to add a closure relation to obtain a macroscopic equation (that is, only with variables 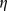 and 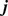). For situations when the distribution is close to isotropic, 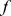 may be approximate by the first terms of its Fourier expansion. In that case,
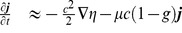
(17)


Considering furthermore that the temporal variation of 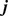 is negligible over the other terms (diffusion approximation), the diffusion equation holds:
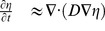
(18)where the diffusion coefficient 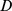 is:
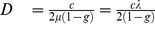
(19)


Starting from a location 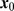 at time 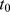, an ant obeying such a diffusion process in an infinite medium would spread from 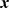 following an isotropic spatial probability density with a spatial variance 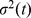, depending on 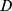. If 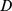 is uniform the probability density is given by:
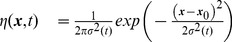
(20)with 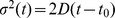 meaning that the variance of the normal distribution increases linearly with time.

The corresponding displacement 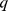 from 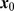 to 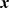 would then follow a probability density 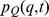:
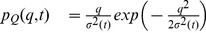
(21)Its second moment, the Mean Square Displacement, naturally increases linearly with time as well, following:
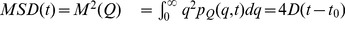
(22)


The Mean Square Displacement is then a measure of the spatial spreading of the ant over time.

In cases when speed varies with time, it can be computed as a function of the number of reorientations events 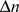 rather than time, following [65]:
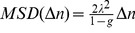
(23)


For more formal developments, see for example [29], [68]–[70].

## Supporting Information

Movie S1 **Example of ant walking down on the canvas.** The ant was gently picked up with a small pig hair paintbrush, the brush head touched the canvas at the center point and the ant was left to walk down from the brush onto it. In this particular case, the ant took approximately seven minutes to walk down from the brush (hence, some part of the movie has been cut), but they usually made it in approximately one minute.
(MOV)

Movie S2 **Short recording of a typical tracking session.** The program tracks the location of the ant within a 40×40 pixels square centered around its location in the previous frame. Filtering and thresholding the background color yields a binary representation of this area, with white pixels corresponding to the ant and background speckles. A partition algorithm detects the largest spot, from which the centroid is extracted (red dot).
(MOV)

Figure S1 **Distribution of path lengths and angular deviations on artificial data.** Statistics for the artificial trajectory are shown in blue, estimations from the segmentation algorithm are shown in orange, and theoretical distributions are shown in dark pink. The upper panel shows the distribution of segments lengths, with the cumulative distribution on the left, and the survival function on the right. The lower panel shows the distribution of angular deviations between segments, with the cumulative distribution on the left, and the polar histogram on the right. For the latter, the three histograms have been scaled differently for comparison purposes.
(TIFF)

Figure S2
**Sensitivity analysis of the segmentation algorithm using artificial data.** Estimated values of 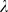 and 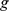 are shown as a function of the value 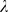 used to generate 300 artificial trajectories (for each point) under the model hypothesis, and 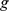 fixed to 0.6. On the left, the tracking noise was fixed to the noise estimated from the data, 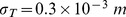 and the segmentation criterion 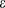 (MAE, Maximal Accepted Error) was varied from 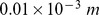 to 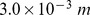. For values close to the finally chosen criterion 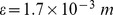 (red lines), the segmentation procedure returns a fair estimation of both 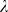 and 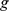, and more importantly in the present context, captures almost perfectly the varying 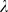. On the right, the same is true for a fixed value of the MAE, and varying the noise, so the results are also robust against a rough estimation of the tracking noise.(TIFF)

Dataset S1
**Trajectories for** 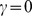 **.**
(ZIP)

Dataset S2
**Trajectories for** 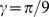 **.**
(ZIP)

Dataset S3
**Trajectories for** 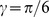 **.**
(ZIP)

Dataset S4
**Trajectories for** 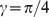 **.**
(ZIP)

Dataset S5
**Trajectories for** 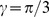 **.**
(ZIP)
