# Supplementary figures and images for: How Do Ants Make Sense of Gravity? A Boltzmann Walker Analysis of Lasius niger Trajectories on Various Inclines
Source: PLoS One. 2013 Oct 29;8(10):e76531. doi: 10.1371/journal.pone.0076531 (PMC3812222; doi:10.1371/journal.pone.0076531)

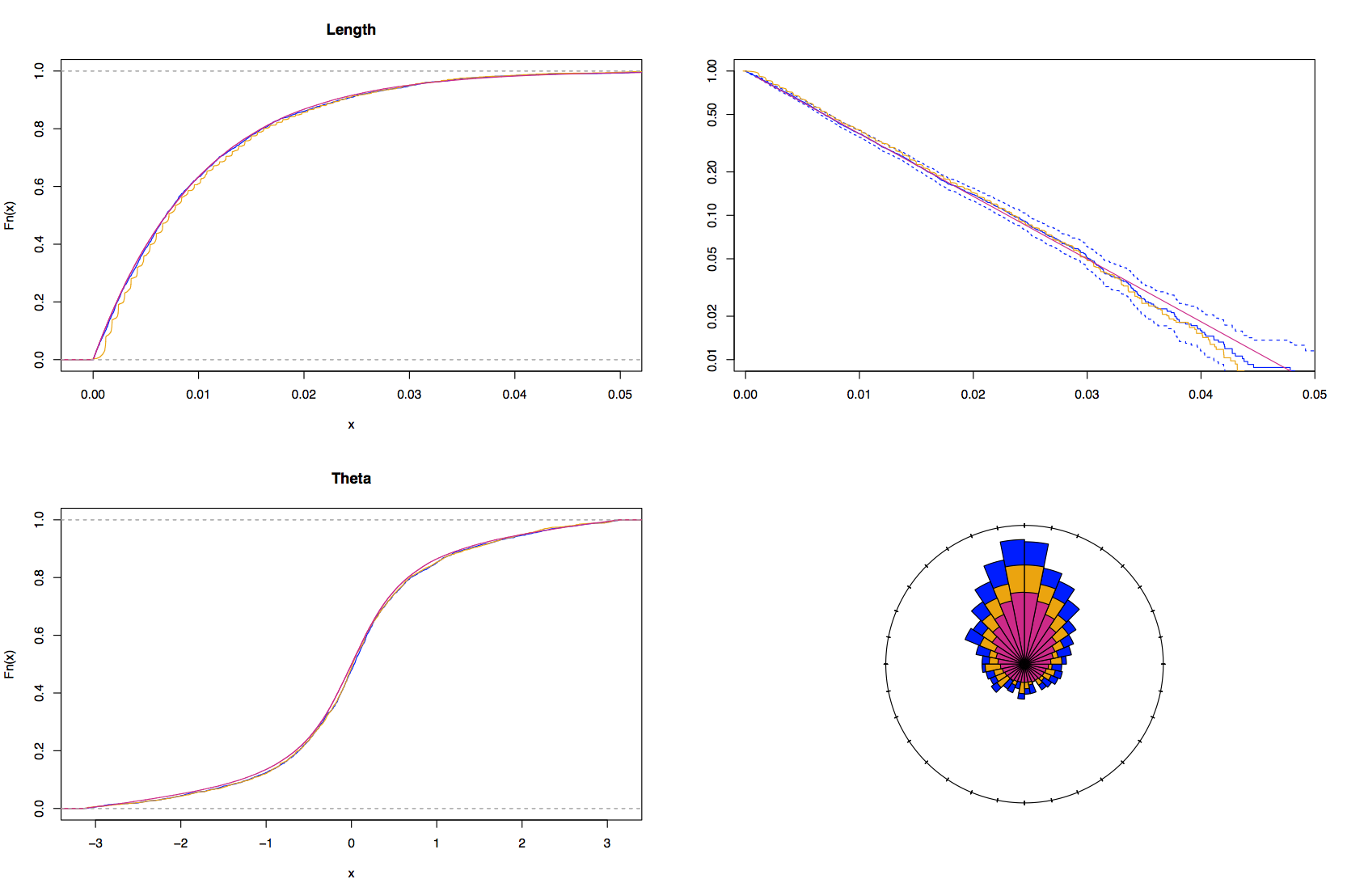

Supplement: Figure S1 — Distribution of path lengths and angular deviations on artificial data. Statistics for the artificial trajectory are shown in blue, estimations from the segmentation algorithm are shown in orange, and theoretical distributions are shown in dark pink. The upper panel shows the distribution of segments lengths, with the cumulative distribution on the left, and the survival function on the right. The lower panel shows the distribution of angular deviations between segments, with the cumulative distribution on the left, and the polar histogram on the right. For the latter, the three histograms have been scaled differently for comparison purposes. (TIFF) [file pone.0076531.s003.tiff]

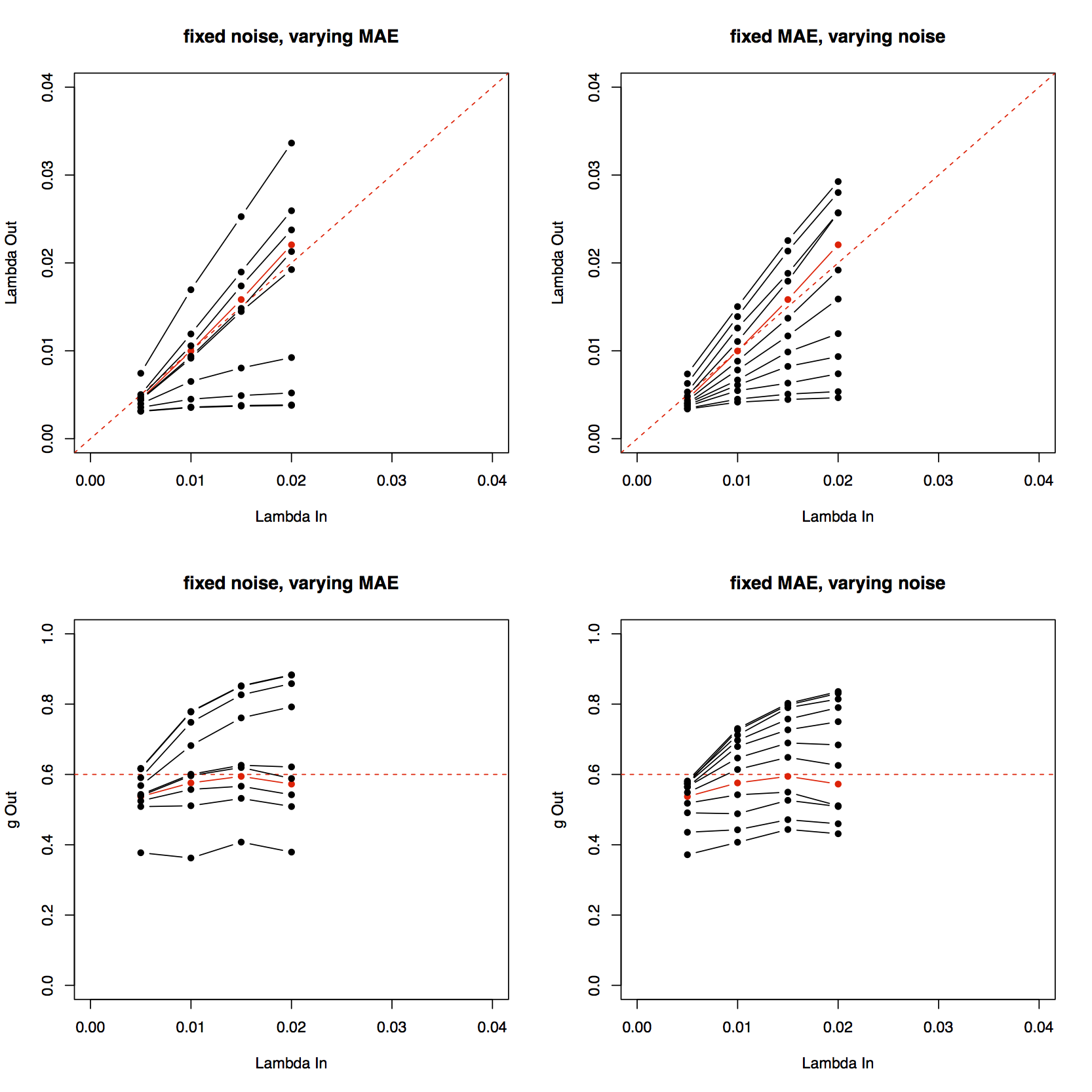

Supplement: Figure S2 — Sensitivity analysis of the segmentation algorithm using artificial data. Estimated values of and are shown as a function of the value used to generate 300 artificial trajectories (for each point) under the model hypothesis, and fixed to 0.6. On the left, the tracking noise was fixed to the noise estimated from the data, and the segmentation criterion (MAE, Maximal Accepted Error) was varied from to . For values close to the finally chosen criterion (red lines), the segmentation procedure returns a fair estimation of both and , and more importantly in the present context, captures almost perfectly the varying . On the right, the same is true for a fixed value of the MAE, and varying the noise, so the results are also robust against a rough estimation of the tracking noise. (TIFF) [file pone.0076531.s004.tiff]
